# The Solicitor's Statement of Facts

**Published:** 1886-12-11

**Authors:** W. Capel Slaughter


### THE SOLICITOR'S STATEMENT OF FACTS.

The following are the facts with regard to the premature and unauthorised publication of this article in *The Globe* of Wednesday, the 24th November, and, excepting the portion printed in italics, they are admitted by *The Globe* to be correct:—

On the 23rd November the Editor of The Hospital, writing to the other leading journals, wrote the Editor of *The Globe*, with the "item of news," as to Lord Salisbury having contributed the article to The Hospital. This letter was received on the 24th November.

A representative of *The Globe* at once called at the private residence of The Hospital Editor, and learning that he was out, showed the servant the Editor's signature to the letter above referred to, and then asked for, and obtained, the name and address of the publishers of The Hospital. *The Globe* representative then went at once to the publishers, showed the Editor's letter as before, and, stating that he had come from the Editor of *The Globe, induced the publishers to believe that he had the authority of the Editor of* The Hospital *to receive a copy of the article*, which was given him accordingly, with the result that it was published in *The Globe* the same afternoon.

The correspondence with *The Globe*, submitted to counsel, affords the proof of these facts, and the printer's letter is proof of that portion of them disputed by *The Globe*.

W. CAPEL SLAUGHTER.

